# Use of validated objective methods of locomotion characteristics and weight distribution for evaluating the efficacy of ketoprofen for alleviating pain in cows with limb pathologies

**DOI:** 10.1371/journal.pone.0218546

**Published:** 2019-06-18

**Authors:** Maher Alsaaod, Mahmoud Fadul, Ramona Deiss, Esther Bucher, Juergen Rehage, Jacopo Guccione, Adrian Steiner

**Affiliations:** 1 Clinic for Ruminants, Vetsuisse-Faculty, University of Bern, Bern, Switzerland; 2 Department of Surgery and Anaesthesia, Faculty of Veterinary Medicine, University of Khartoum, Khartoum North, Khartoum, Sudan; 3 Department of Veterinary Medicine and Animal Productions, University of Napoli Federico II, Napoli, Italy; 4 Clinic for Cattle, University of Veterinary Medicine, Hannover, Germany; University of Illinois, UNITED STATES

## Abstract

In veterinary practice pain alleviation plays a part in managing lameness. The aim of this randomized and placebo-controlled clinical study was to evaluate the effect of a single administration of ketoprofen on locomotion characteristics and weight distribution in cattle with foot (located up to and including the fetlock; *n = 31*) and (proximal to the fetlock; *n = 10*) pathologies. Cattle were randomly allocated to either the ketoprofen (group K; intravenous 3 mg/kg of body weight; *n* = 21) or an equivalent volume of isotonic sterile saline solution (group P; *n* = 20). Two accelerometers (400 Hz; kinematic outcome = stance phase duration; kinetic outcome = foot load and toe-off), a 4-scale weighing platform (weight distribution and SD of the weight) and a subjective locomotion score were measured before (baseline) and after 1 h and 18 h of treatment. All variables were expressed as differences across contralateral limbs, and the measurements at 1 h and 18 h were compared to the baseline. A repeated measures ANOVA was used to determine the differences between groups K and P. A logistic regression model with a binary outcome (0 = no improvement and 1 = improvement of the differences across the contralateral limbs over time) was calculated. Mean (± SD) of locomotion scores at baseline were not significantly different (*P* = 0.102) in group K (3.10 ± 0.80) as compared to group P (3.48 ± 0.64). Cattle of group K showed significantly lower differences across contralateral limbs at 1 h as compared to group P for the relative stance phase and the weight distribution. Only the treatment (P versus K) remained a significant factor in the model for relative stance phase (odds ratio (OR) = 6.5; 95% CI = 1.38–30.68) and weight distribution (OR = 6.36; 95% CI = 1.30–31.07). The effects of ketoprofen were evident in improving the differences across contralateral limbs—both for stance phase during walking and weight bearing during standing—after 1 h but not after 18 h of administration.

## Introduction

Pain management is a public concern and a key component in assessing and improving animal welfare [1, 2]. Therefore, alleviating pain is becoming an increasingly important consideration in routine veterinary practice [3, 4]. A prerequisite for alleviating pain is early recognition [5]. Inadequate ability to assess and recognize pain in cattle remains a major problem [6–8]. Cattle are a prey species with a stoic character, rarely showing overt signs of pain until the stimulus is severe and the disease in an advanced state [9]. Previous studies showed that cattle practitioners, as well as claw trimmers and farmers, might underestimate the sensitivity towards pain in cattle compared to other species [10, 11]. Additionally, early culling and euthanasia rates due to lameness were increased in order to terminate suffering from pain [12, 13].

Foot pathologies induce an hyperalgesic state and produce a range of inflammatory mediators at the site of the pathology, including the release of prostaglandins, thromboxanes and leukotrienes [14]. Nonsteroidal anti-inflammatory drugs (**NSAIDs**) such as tolfenamic acid, meloxicam, flunixin meglumine and ketoprofen have been administered to relieve pain and to reduce inflammation in cattle [15–19]. Ketoprofen is a propionic acid derivative that inhibits the cyclo-oxygenase (COX-1 and COX-2) enzymes, and this in turn leads to reduced peripheral and central prostaglandin production [20].

The analgesic effect of ketoprofen in lame cows has already been explored during the treatment of lameness. Thomas et al. [19] found that lame cows treated for a claw horn lesion with a therapeutic trim, block, and the NSAID ketoprofen were more likely to recover to a sound locomotion score than those treated with a therapeutic trim only. Ketoprofen was shown to moderately reducing hyperalgesia of lame cows in the recovery period after therapeutic claw trimming [16], improving the weight distribution between sound and lame legs [21], decreasing the weight shifting across the rear legs [22].

Although these studies in general showed a moderate pain mitigating effect of ketoprofen, they do not allow us to objectively determine the short-term effect of ketoprofen on cows' gait. Moreover, the newly developed tools for objectively assessing the cows' gait represent a promising approach to detect cows with foot pathologies and even very slight cases of lameness [23–25]. Therefore, the aim of this study was to evaluate the effect of the NSAID Ketoprofen (Rifen Streuli AG, Switzerland) during the time period when ketoprofen is thought to be active (24 h) in cows affected with limb pathologies using gait scoring and validated automated tools of weight bearing and gait analysis. We hypothesized that (i) a single administration of ketoprofen improves the cows' gait within 24 h of ketoprofen administration as compared with cows that received a placebo; (ii) using the combination of validated tools for objective lameness evaluation is appropriate to detect the short-term effect of ketoprofen.

## Materials and methods

### Ethical statement

The study protocol was approved by the animal experimentation committee of the canton of Berne, Switzerland (permission # 25601).

### Animals, selection and clinical examination

The study was carried out between January 2015–November 2017 at the Clinic for Ruminants, Vetsuisse-Faculty, University of Bern. The cows were referred to the clinic and submitted to a thorough orthopedic examination [26], including a radiographic and ultrasonographic examination if indicated. In case of involvement of synovial structures, macroscopical, cytological and microbiological synovial fluid analysis was performed. Final diagnoses are given in Table 1.

**Table 1:** Clinical orthopaedic findings in cattle included in the study. The clinical cases were randomly allocated to either the ketoprofen (group K) or placebo group (group P) and further categorized into foot (located up to and including the fetlock) vs. (proximal to the fetlock). The categories were included as independent variables of logistic regression model for ketoprofen treatment. The limb pathologies were arranged from distal to proximal location.

	Foot location	Location proximal to the fetlock
Pathological findings	Vertical horn wall fissure (*n =* 1), horn bruise (*n =* 1), WLD (*n =* 3), SU (*n =* 1), laceration of the interdigital space (*n =* 1), fracture of P3 (*n =* 1), osteitis of P3 (*n =* 9), aseptic arthritis of DIJ (*n =* 1), septic arthritis of DIJ (*n =* 2), septic arthritis of the PIJ (*n =* 3), osteoarthritis of the PIJ (*n =* 1), traumatic periarthritis of the fetlock joint (*n =* 1), septic arthritis of the fetlock joint (*n =* 3), arthrosis of the fetlock joint (*n =* 1), tendovaginitis of CDFTS (*n =* 2).	Epiphysitis of the distal metacarpus (*n =* 1), laceraton of the metatarsus with bone sequestration (*n =* 1), middle to high degree bone spavin (*n =* 1), septic arthritis of the tarsucrural joint (*n =* 2), epiphysitis of the distal radius (*n =* 1), physitis of the distal radius and ulna (*n =* 1), septic arthritis of the elbow joint (*n =* 1), septic arthritis and physitis of the distal femur (*n =* 1), fracture of the ileum (*n =* 1).

(WLD) white-line disease

(SU) sole ulcer

(DIJ) distal interphalangeal joint

(PIJ) proximal interphalangeal joint

(CDFTS) common digital flexor tendon sheath

Informed consent was obtained from the animal owners for the use of their animals in this study. A total of 41 cattle with unilateral limb pathology (fore or hind limb) were included in this study. Furthermore, cattle must not have exhibited any relevant systemic concentration of analgesics at the beginning of the study. “Cattle with no relevant systemic concentration of analgesics” was defined as those animals without any analgesic pretreatment within the previous 2 months or such with an analgesic pretreatment that was ceased earlier than 4.5 x the elimination half-life of the respective compound.

Age of cattle (mean ± SD) was 53.3 ± 31.2 months with a mean body weight of 562.1 ± 153.2 kg. The study group included 5 males and 36 females. Breeds were Holstein Friesian (*n* = 10), Eringer (*n* = 9), Swiss Fleckvieh (*n* = 8), Red Holstein (*n* = 7), Brown Swiss (*n* = 4), Simmental (*n* = 2) and Jersey (*n* = 1). The full description of the used cattle can be found in S1 Dataset.

### Experimental procedures and treatments

The study was performed as a double-blinded, randomized, and placebo-controlled clinical trial. Both, persons who administrated the treatments and persons performing data processing including all statistical analyses were blinded as to the treatment groups. Cattle were randomly allocated within 24 h after final diagnosis to either the ketoprofen (**group K**; *n* = 21) or placebo group (**group P**; *n* = 20), receiving one dose of ketoprofen (3 mg/kg of BW i.v.; Rifen Streuli Pharma AG, Switzerland, http://www.streuli-pharma.ch/) or an equivalent volume of sterile isotonic saline solution (i.v., NaCl 0.9% steril Laboratorium Dr. G. Bichsel, Interlaken-Switzerland), respectively. The choice of treatment for 2 consecutive experimental animals was done by drawing a lot from a pot containing one lot of each treatment group. Data of locomotion characteristics and weight distribution were collected and recorded for further analysis at three data collection time points: immediately before treatment (baseline; **T**_**0**_), 1 h after treatment (**T**_**1**_) and 18 h after treatment (**T**_**18**_). Between baseline and T_1_ and between T_1_ and T_18_, cattle were housed in a confined free stall cubicle of 3x3 m, kept on rubber mats covered with deep straw bedding and milked and fed according to standards of good farming practice.

### Data collection

#### Recording and analysis of locomotion variables

**Clinical gait score assessment:** All cows were videotaped using a digital video camera (50 frames/s; Sony HDR-PJ740VE, Sony Corporation, Tokyo, Japan) to record the locomotion while the respective cow was walking on an asphalt floor in a straight line for 20 m. The video recordings were blinded as to group allocation and pathology, and locomotion was scored using a 1 to 5 numerical rating system (**NRS**) with 0.5-point increments (where 1 = non-lame and 5 = severely lame) based on 6 specific gait attributes (back arch, head bob, tracking up, joint flexion, asymmetric steps and reluctance to bear weight) [27]. To maximize the reliability of clinical locomotion scoring, the mean value of the scores given by 3 trained independent veterinary specialists was calculated and used for further analysis.

**Measurement of gait cycle variables:** At each time point (T_0_, T_1_ and T_18_), immediately before data recording, cows were equipped with two stand-alone 3D accelerometers (400 Hz; USB Accelerometer X16-4; Gulf Coast Data Concept, Waveland, USA), which were fitted at the level either of both metatarsi or both metacarpi, depending on the location of the pathology. The gait cycle variables (cow pedogram variables) were extracted, using the validated Cow-Gait-Analyzer as described by Alsaaod et al. [28]. The pedogram variables comprised of temporal events of kinematic outcomes (relative stance and swing-phase durations) and peaks of kinetic outcomes (foot load, toe off) (Table 2).

**Table 2:** Definitions of gait cycle variables (cow pedogram; temporal events [kinematic outcome = relative stance phase] and peaks [kinetic outcome = foot load, toe-off]) extracted by use of the Cow-Gait-Analyzer as described by Alsaaod et al. [28] and the 4-scale weighing platform variables (Mean weight distribution and SD_weight_) as described by Nechanitzky et al. [24].

Method	Item	Variable	Definition
Cow pedogram	Kinematic (temporal)	Stance phase (%)	Percentage proportion of time that the claw is in contact with the ground to the total gait cycle duration
	Kinetic (peak)	Foot load (g)	Maximum acceleration (peak) of the initial ground contact of the claw
		Toe-off (g)	Maximum acceleration (peak) of the termination of the ground contact of the tip of the claw
4-scale weighing platform	Kinetic (temporal)	Mean weight (kg)	Mean weight applied on each limb
		SD_weight_ (kg)	Standard deviation of the weight applied on each limb

**Weighing platform:** Weight distribution across contralateral limbs was measured while cows were standing on a 4-scale weighing platform (1.94 × 1.06 m; ITIN & HOCH GmbH, Fütterungstechnik, CH-4410 Liestal, Switzerland). The platform consisted of 4 recording units (0.78 × 0.55 m), with one hermetically sealed load cell each (HBM, Hottinger Baldwin Messtechnik AG, Volketswil, Switzerland) and covered with individual rubber mats of 1-cm thickness as described by Nechanitzky et al. [24]. Cattle were given 5–10 min to get used to standing quietly on the balance. When they were standing with each limb positioned on the appropriate unit, the weight measurement was started manually. Total data collection time was five minutes at a frequency of 10 Hz. During this time, cattle were closely observed, and if there was defecation or urination, the measurement was stopped and restarted. Data collection was automatically stopped as soon as the measured total weight deviated by more than 5% from the originally measured total body weight of the cow. The mean weight and the standard deviation of the weight (**SD**_**weight**_) applied to each limb were calculated (Table 2).

### Data analysis and statistics

Data analysis was performed at the cow level, and all included variables that represented the difference across the contralateral limbs of the affected limb pair, the differences between T_0_ and T_1_ (**ΔT**_**1**_) and between T_0_ and T_18_ (**ΔT**_**18**_) were used for the final analysis. The power of the study for the sample size of 41 cows was calculated before the conduction of the statistical analysis, based on setting the effect size of 0.6 and a confidence level of 5%, using an online calculator (https://www.anzmtg.org/stats/PowerCalculator/PowerTtest). The effect size (*d* = 0.6) was calculated as an absolute difference of the means of locomotion scores of group K versus group P divided by the standard deviation, considered according to Cohen [29] as a medium size effect. The power of the study was estimated at 0.76, which was considered to be high enough for applying one-way Analysis of Variance (**ANOVA**).

The normality of gait variables was checked with the Shapiro-Wilk test and a natural logarithm was calculated for normality data distribution (Shapiro-Wilk test statistic for all variables was >0.95). The variables were analyzed using the software package NCSS^10^ (NCSS LLC, Kaysville, UT) (S1 and S2 Datasets). An ANOVA was performed to compare between group K and group P for ΔT_1_ and ΔT_18_ separately. All variables including locomotion scores were considered as "continuous variables" to perform the ANOVA. A Bonferroni corrected *P*-value was calculated; the significance value was set at *P* ≤ 0.05 (without Bonferroni adjustment). Only the significant variables of pedogram and weight distribution were included in the multivariable analysis to build up the binary outcome. The binary outcome of the model represented no improvement when the difference across contralateral limbs was equal or increased (0) and improvement, when the difference across contralateral limbs decreased (1) at T_1_ or T_18_ as compared to T_0_. The independent variables included the following categories: Treatment (group K vs. group P); NRS at the baseline measurement (NRS ≥ 3 vs. NRS < 3); limb pathology location (foot = located up to and including the fetlock) vs. (proximal to the fetlock) and the disease duration (< 2 weeks vs. ≥ 2 weeks). The model-building strategy was to initially include individually each parameter with *P*<0.25 in the univariable analysis in the models. This was followed by a stepwise forward selection with elimination of all non-significant parameters. Model fit was assessed by the Hosmer and Lemeshow test and a visual assessment of residuals. The model output was represented as odds ratio (**OR**) with confidence interval (**CI**) (95%) and the significance level was based on α ≤ 0.05.

Two multivariate linear regression analyses were performed to investigate whether the NRS is a confounder for the relative stance phase and weight distribution at T_0_, respectively, with the NRS and ketoprofen treatment as independent variables.

The change of the stance and swing phases (difference value across the contralateral limbs) is analogous. Therefore, the statistical analyses for both variables were performed, but only the relative stance phase was reported. The gait cycle variables of the cow pedogram were calculated as the absolute difference across the contralateral limbs. The weight distribution was calculated as the percentage absolute difference of the mean weight across the contralateral limbs (**Δ**_**weight**_ **(%)**):

$$\Delta weight\;(\%)=\frac{mean\;weight\;applied\;on\;healthy\;limb-mean\;weight\;applied\;on\;limb\;with\;pathology\;}{mean\;weight\;applied\;on\;healthy\;limb+mean\;weight\;applied\;on\;limb\;with\;pathology}\;x\;100$$

All parameters of gait score, gait cycle and weight scale were blinded as to the cows, time of measurements (T_0_, T_1_ and T_18_) and treatment group (group K and P).

## Results

### Effect of ketoprofen on gait, gait cycle, and weight distribution

Data of various gait and weight variables at each time point are given in Table 3. At ΔT_1_ only the relative stance phase duration (mean ± SD; 3.85 ± 5.77 (%) and -1.51 ± 2.94 (%)) and the weight distribution (7.76 ± 7.95 (%) and 0.827 ± 8.6 (%)) showed significant differences between group K and group P (*P* = 0.001 and 0.002), respectively. Group K had significantly lower differences across the contralateral limbs at ΔT_1_ as compared to group P. However, neither the gait variables nor the weighing platform variables showed differences between group K and P at ΔT_18_ (Table 3).

**Table 3:** Mean (±SD) of gait cycle and weighing platform variables of ketoprofen group (K) versus placebo group (P) at baseline (T_0_), ΔT_1_ (the differences between (T_0_) and after 1 h (T_1_) of ketoprofen administration) and ΔT_18_ (the differences between (T_0_) and after 18 h (T_18_) of ketoprofen administration). The gait cycle variables and the 4-scale weighing platform variables were calculated as the difference across the contralateral limbs.

Item	T_0_					ΔT_1_					ΔT_18_				
	Group K		Group P		*P*-value	Group K		Group P		*P*-value	Group K		Group P		*P*-value
	mean	^1^ SD	mean	SD		mean	SD	mean	SD		mean	SD	mean	SD	
Stance phase (%)	10.3	9.05	13.7	12.5	0.338	^2^ 3.85	5.77	^3^ -1.51	2.94	0.001	-0.253	6.29	1.06	9.38	0.612
Foot load (*g*)	4.01	4.67	8.20	7.50	0.043	0.634	3.24	1.81	5.31	0.411	-0.264	3.80	2.75	10.5	0.247
Toe-off (*g*)	3.01	2.0	2.71	2.25	0.756	0.188	0.988	-0.02	1.09	0.532	-0.149	1.64	0.905	6.07	0.469
Δ_weight_ (%)	44.0	30.4	50.4	28.0	0.490	7.76	7.95	-0.827	8.60	0.002	0.753	9.37	-4.58	20.9	0.293
SD_weight_ (kg)	0.889	0.177	0.927	0.202	0.523	-2.82	9.32	0.609	4.86	0.151	-1.11	10.2	4.40	11.2	0.108
Gait score	3.10	0.8	3.48	0.640	0.102	0.190	0.432	0.075	0.335	0.347	-0.119	0.445	-0.05	0.583	0.673

^1^ SD: standard deviation

^2^ the positive value indicates an improvement in the difference across the contralateral limbs (T_0_ is higher than T_1_)

^3^ the negative value indicates a worsening in the difference across the contralateral limbs (T_0_ is lower than T_1_)

### Gait score and its correlation to relative stance phase and weight distribution

Mean (± SD) of locomotion scores at T_0_ (baseline) were 3.10 ± 0.80 in group K and 3.48 ± 0.64 in group P. Gait scores were not significantly different between groups at T_0_ (*P* = 0.102). The gait scores were highly correlated with differences across the contralateral limbs for relative stance phase durations (*r* = 0.73) and moderate for weight distribution (*r* = 0.5).

### Logistic regression for ketoprofen treatment

Only the variables of “relative duration of the stance phase” and “weight distribution” (Δ_weight_ (%)) were included separately in each multivariable analysis to perform the binary outcome (improvement or worsening of the differences across contralateral limbs) at ΔT_1_. The logistic regression showed that differences across contralateral limbs for the relative duration of the stance phase and the weight distribution at ΔT_1_ were both associated with treatment. Administration of a single dose of ketoprofen had a significant effect on improving the differences across the contralateral limbs for the relative stance phase (regression coefficient = 1.87; *P* = 0.018; OR = 6.5; 95% CI = 1.38–30.68) and weight distribution (regression coefficient = 1.85; *P* = 0.022; OR = 6.36; 95% CI = 1.30–31.07) as compared to group P. The categories of gait score, pathology location and duration of limb pathologies were not significantly associated with the binary outcome of the logistic regression model.

The multivariate linear regression analysis showed that the NRS did not confound the effect of ketoprofen at T_0_ for both relative stance phase duration and weight distribution (*P* = 0.971 and *P* = 0.986), but the effect was still significant for ketoprofen (*P* = 0.019 and *P* = 0.018), respectively.

## Discussion

Ketoprofen has been approved for use in lactating cows in the European Union and Canada, with a milk withdrawal period of 0 days. Therefore, ketoprofen is a valuable option for cattle practitioners to relieve pain and inflammation in lactating dairy cows. To the best of the authors' knowledge, this is the first study that investigates the effects of ketoprofen on locomotion characteristics and weight bearing within the time period of 24 h, when ketoprofen is thought to be active. The effects of ketoprofen were apparent in improving the differences across contralateral limbs—both for stance phase duration during walking and weight bearing during standing– 1 h after administration; this improvement was, however, no longer present after 18 h.

The plasma half-life of ketoprofen is less than 2 h (in adult cattle it is 0.42 h), the time period when ketoprofen is thought to be effective is 24 h, and within this period, 80% of the dose is eliminated in the urine [30, 31]. Consequently, Whay et al. [16] et al. reported that the period of 1 h after administration in lame cows was theoretically sufficient for ketoprofen to become active at the inflammatory site, although this was not confirmed by the results of the nociceptive threshold test. Therefore, the analgesic was administered once and evaluated objectively after 1 h and 18 h (time periods when ketoprofen is thought to be active) in the current study. Under practice conditions, repeated applications of ketoprofen are required to maintain adequate analgesic concentrations over several days [30]. In accordance with this, Whay et al. [16] reported a significant modulation of the cows’ hyperalgesia after the use of ketoprofen for 3 days to treat foot lesions associated with lameness as compared to a control group.

In order to objectively assess the effect of ketoprofen alone, treatment of limb pathologies was not performed concurrently with the assessment of ketoprofen. Furthermore, cattle included in our study did not receive any pretreatment with an NSAID within less than 4.5 times the elimination half-life of the respective compound from T_0_. Previous studies showed that acute lesions caused shorter periods of hyperalgesia as compared to chronic lesions [14, 16, 32]. In our study, we included cows with various limb pathologies and different degrees of duration. There was neither any effect of disease duration nor of type of foot pathology on gait variables that might indicate the selective use of ketoprofen in specific cases.

The gait score was highly correlated to the relative stance phase duration during walking and moderately to weight distribution during standing. This provides evidence that both the subjective and the objective methods are useful to evaluate the gait during walking.

The locomotion score was considered as continuous and not as categorical (lame vs. non-lame) variable in the current study. We assumed that the categorical form may be relevant for the classification of the lameness status (lame vs. non-lame), while this is not the focus of our study. The locomotion score was rather considered as an effect variable for evaluating the efficacy of ketoprofen, justifying its use as a continuous variable. The not statistically significant difference of locomotion scores between cows of groups K and P at T_0_ (results from multivariate linear regression analysis) indicates that the random allocation of the cows to the treatment groups was well balanced concerning the initial locomotion score. However, this does not completely rule out the possibility that locomotion score at T_0_ confounds the results of ketoprofen treatment.

An effect of ketoprofen on locomotion scores was not found, supporting the results of previous studies. Whay et al. [16] reported that the lameness score was a less sensitive indicator than the nociceptive threshold test to determine the effect of ketoprofen, although a modulation in hyperalgesia in lame cows was observed. Flower et al. [21] reported a moderate reduction of the NRS (0.25 units) after ketoprofen treatment for 3-days. The effect was similar to the reduction of the NRS reported by Rushen et al. [33] (0.3 units) after injecting the bulbs of the affected limb with lidocaine and the results of our study with a non-significant improvement of 0.2 units. In general, the objective assessment of gait, weight bearing and activity has been shown to be more sensitive than gait scores as a method of evaluating the effect of ketoprofen [34]. Moreover, locomotion score is not sensitive enough to detect a slight gait alteration [35, 36]. Therefore, one of the benefits of objective lameness assessment is to detect slight gait alterations which are not visible to the trained observer.

A combination of stance phase duration at walking and weight distribution at standing was the best combination of parameters to determine the effect of ketoprofen. Rushen et al. [33] measured weight bearing using force plates and found a more even weight distribution among the limbs when the cows were administrated a local anaesthesia. A follow-up study by Chapinal et al. [34] showed a decrease of the SD of the weight applied to the hind limbs on the days when ketoprofen injections were given compared to the days before and after treatment for both lame and non-lame cows (18% and 12%, respectively), while no decrease was detected in the front limbs. In comparison with the previous study by Chapinal et al. [34], both cows with front and hind unilateral limb pathologies were included and the short-term effect of ketoprofen was determined. Novak et al. [37] evaluated in a placebo-controlled study the weight shifting of lame cows treated twice with ketoprofen administered intramuscularly with an interval of 48 h. They reported a significant decrease of the weight shifting across hind limbs at 6 and 12 h of the first treatment while no effect was observed at 2, 24 or 48 h. The second treatment of ketoprofen decreased the weight shifting at 12 and 24 h. In our study, the SD_weight_ of all limbs was not changed by ketoprofen treatment at any time point of measurement. The variable SD_weight_ is a measure of leg load variability or weight shifting [33]. In a previous study by Nechanitzky et al. [24], it was reported that SD_weight_ is weakly correlated with Δ_weight_ and negatively correlated with locomotion score of lame cows, and thus, slight lameness may be accompanied by more weight shifting as compared with severe lameness. Therefore, Δ_weight_ is a more reliable parameter than SD_weight_ to determine the degree of pain associated with an orthopaedic problem.

Only the differences of the parameters stance phase duration and weight bearing across contralateral limbs at 1 h after administration of ketoprofen were associated with improvement as compared to the saline group. Ketoprofen was reported as rapidly exiting the bloodstream to enter the tissue compartment at the site of inflammation, where it is pharmacologically active [38]. Flunixin meglumine is approved for use in cattle and often used as extra-label drug for pain relief in cattle [39]. A placebo-controlled study by Chapinal et al. [34] reported that administering flunixin meglumine to lame and non-lame dairy cows at the time of hoof trimming had no effect on gait score or any measures of weight distribution, while Wagner et al. [18] reported that lame cows treated with flunixin meglumine intravenously showed a significant decrease of the weight-shifting across hind limbs at 6, 12, and 24 h but not at 2 h after treatment compared with the placebo group, providing evidence that flunixin meglumine alleviates orthopedic pain in lactating dairy cows. It must be mentioned that treatment of painful orthopaedic disorders should not be restricted to administration of NSAIDs but focus on treatment of the cause of lameness. Otherwise, deterioration of the primary disease and overload of the affected limb will be supported.

## Conclusions

A single administration of ketoprofen significantly reduced the differences across the contralateral limbs at walking and standing after 1 h of administration, but this effect was not detectable after 18 h. The results of this study reveal that measuring stance phase duration while cows are walking and weight bearing across contralateral limbs while standing shows great potential as an automated method of evaluating the effect of NSAIDs on the musculoskeletal apparatus of lame cattle.

## Supporting information

S1 Dataset Animals, cases and statistical analysis Excel data. Data used for statistics.
(XLSX)

S2 Dataset Statistical analysis NCSS. Data used for statistics.
(NCSS)
