# Correction: Use of validated objective methods of locomotion characteristics and weight distribution for evaluating the efficacy of ketoprofen for alleviating pain in cows with limb pathologies

**DOI:** 10.1371/journal.pone.0225565

**Published:** 2019-11-14

**Authors:** Maher Alsaaod, Mahmoud Fadul, Ramona Deiss, Esther Bucher, Juergen Rehage, Jacopo Guccione, Adrian Steiner

The affiliation for the sixth author is incorrect. Jacopo Guccione is not affiliated with #4 but with #3: Department of Veterinary Medicine and Animal Productions, University of Napoli Federico II, Napoli, Italy.
